# High-Quality Genome Assembly of *Eriocheir japonica sinensis* Reveals Its Unique Genome Evolution

**DOI:** 10.3389/fgene.2019.01340

**Published:** 2020-01-17

**Authors:** Boping Tang, Zhongkai Wang, Qiuning Liu, Huabin Zhang, Senhao Jiang, Xinzheng Li, Zhengfei Wang, Yue Sun, Zhongli Sha, Hui Jiang, Xugan Wu, Yandong Ren, Haorong Li, Fujun Xuan, Baoming Ge, Wei Jiang, Shusheng She, Hongying Sun, Qiang Qiu, Wen Wang, Qun Wang, Gaofeng Qiu, Daizhen Zhang, Yongxin Li

**Affiliations:** ^1^ Jiangsu Key Laboratory for Bioresources of Saline Soils, Jiangsu Provincial Key Laboratory of Coastal Wetland Bioresources and Environmental Protection, Jiangsu Synthetic Innovation Center for Coastal Bio-agriculture, Yancheng Teachers University, Yancheng, China; ^2^ Center for Ecological and Environmental Sciences, Northwestern Polytechnical University, Xi'an, China; ^3^ Institute of Oceanology, Chinese Academy of Sciences, Qingdao, China; ^4^ National Engineering Laboratory of Marine Germplasm Resources Exploration and Utilization, Zhejiang Ocean University, Zhoushan, China; ^5^ National Engineering Research Center for Facilitated Marine Aquaculture, Zhejiang Ocean University, Zhoushan, China; ^6^ Key Laboratory of Freshwater Aquatic Genetic Resources, Ministry of Agriculture and Rural Affairs, Shanghai Ocean University, Shanghai, China; ^7^ China-Hong Kong Ecology Consultant Company, Hong Kong, Hong Kong; ^8^ College of Life Sciences, Nanjing Normal University, Nanjing, China; ^9^ Department of Biology, School of Life Science, East China Normal University, Shanghai, China

**Keywords:** *Eriocheir japonica sinensis*, evolution, nanopore, crab, genome assembly and annotation

## Abstract

As an important freshwater aquaculture species in China, the Chinese mitten crab (*Eriocheir japonica sinensis*) has high economic and nutritional value. However, limited genomic information is currently available for studying its basic development and genetic diversity. Here, we performed whole-genome sequencing on Oxford Nanopore Technologies Limited's platform using promethION. The assembled size of *E. j.sinensis* genome was approximately 1.27 Gb, which is close to the estimated size (1.19 Gb). Furthermore, based on assessment using Benchmarking Universal Single-Copy Orthologs (BUSCO) (Simao et al., 2015), 94.00% of the expected eukaryotic genes were completely present in the genome assembly. In addition, repetitive sequences accounted for ~61.42% of the assembled genome, and 22,619 protein-coding genes were annotated. Comparative genomics analysis demonstrated that the Chinese mitten crab diverged from *Penaeus vannamei* ~373.6 million years ago, with a faster evolution rate than shrimp. We anticipate that the annotated high-quality genome of *E. j. sinensis* will promote research on its basic development and evolution and make substantial contributions to comparative genomic analyses of crustaceans.

## Introduction

The Chinese mitten crab, *Eriocheir japonica sinensis* (i.e., *Eriocheir sinensis*), which belongs to the Varunidae family of crustaceans, is a medium-sized burrowing crab containing dense patches of dark setae on its claws and preying on fish, shrimp, snails, mussels, worms, earthworms, and insects (Panning, 1939; Rudnick et al., 2005; Rybczyk et al., 2010). The mitten crab is native to rivers, estuaries, and other coastal habitats of Eastern Asia from North Korea to South China (Gilbey et al., 2008), but has also been introduced to Europe and North America, where it is considered an invasive species (Rudnick et al., 2003; Herborg et al., 2005; Gilbey et al., 2008). These crabs spend most of their life in freshwater, but migrate to shallow estuaries during the breeding season (Bentley, 2011). They mate and deposit eggs at brackish water in late summer and only once in their lifetime, laying eggs at the end of their life cycle (Panning, 1939; Tsukimura, 2008).

Chinese mitten crabs are an economically important and nutritious aquatic food in Asia (Chen and Zhang, 2007; Chen et al., 2007; Jiang et al., 2010). As such, populations have declined drastically due to overfishing, water pollution, and dam construction (Tang et al., 2000; Chang et al., 2008), although resources have been somewhat restored by artificial breeding and release (Jiang et al., 2010; Chen et al., 2015). While their diet (Jin et al., 2003), development (Ng et al., 1998), reproduction (Montú et al., 1996), and immune system (Gai et al., 2009; Zhao et al., 2009) have been studied, a high-quality mitten crab genome has yet to be reported. Importantly, with the rapid development of third-generation sequencing technology, high-quality genomes can now be obtained more easily, which will greatly promote biological research.

In this study, we examined genomic data of *E. j. sinensis* using newly obtained Nanopore long reads and previously made available Illumina short reads. We anticipate that the well-annotated genome and large amount of sequencing data obtained in the current study will promote research on the basic development and evolution of *E. j. sinensis* and also contribute to comparative genomic analysis of related species.

## Materials and Methods

### Sampling, Library Construction, and Sequencing

To obtain high-quality DNA for Chinese mitten crab genome sequencing using Oxford Nanopore Technologies (ONT; Oxford, UK), we sampled one individual (*E. j. sinensis*) from a large vertical lake in Jiangsu, China. The crab was first rinsed under flowing clean water and dissected. After removing the foreign bodies covering its surface, muscle tissue was sampled and ground into powder in liquid nitrogen. DNA was then extracted using a Qiagen Blood & Cell Culture DNA Mini Kit (Germany), with DNA quality checked by agarose gel electrophoresis. The tissue samples of hepatopancreas, muscle, and heart were used for RNA extraction using TRIzol reagent, with RNA concentration and quality assessed using a Qubit fluorometer. The extracted DNA and RNA were sequenced on the Oxford Nanopore PromethION 48 (P48) platform and Illumina NovaSeq 6000 platform, respectively. The genome short reads were downloaded from NCBI (Song et al., 2016) (SRR3081450, SRR3081453, SRR3081454, SRR3081455, and SRR3081456).

### Quality Control of Sequencing Reads

Three different kinds of reads were used for this study, including Nanopore long reads (PromethION 48; Oxford Nanopore; insert size, 20 Kb), Illumina genomic short reads (HiSeq 2000; Illumina), and Illumina RNA-seq reads (NovaSeq 6000; Illumina; insert size, 250 bp). The genomic short reads (81×, produced on Illumina 2000 platform) produced in previous study were downloaded from NCBI: SRR3081450, SRR3081453, SRR3081454, SRR3081455, and SRR3081456. To ensure the reads quality, all low-quality reads, adaptor sequences, and polymerase chain reaction (PCR) duplicates which are the same reads produced by PCR were removed. For the Nanopore long reads, Guppy v2.2.2 was used as ONT basecaller and we only removed the short reads and the low-quality reads. Specifically, for short reads filtering, all the reads longer than 1 Kb were retained by an internal Perl script. For reads quality filtering, for each read longer than 1 Kb were calculated the mean quality value and only reads with mean quality ≥7 were retained using another in-house Perl script. In the RNA sequencing (RNA-seq) data were also removed the low-quality reads, adaptor sequences, and PCR duplicates produced by PCR. After filtering these data, the Illumina reads and Nanopore reads were both aligned to NR database (from NCBI) by BLAST software (blastn) (Altschul, 2012) with *e* value set as 10^−5^. Any reads that had the best blast hit to microorganism were considered as contamination and were removed.

### Genome Characteristic Estimation

To estimate the genome size of the Chinese mitten crab, we implemented the count subprogram of the jellyfish software by 19-mer to generate the binary file which should be the input of the next step histogram to format the kmer histogram using all filtered Illumina short reads. Based on the histogram file, we used GenomeScope v1.0 and made the final estimation. The genome size is calculated by this formula: *G* = *N*/*D*, where the *N* is the total number of 19-mer and the *D* is the average depth of 19-mer.

### Nanopore Long Read Correction and Genome Assembly

The error rate of the Nanopore long reads is much higher than the Illumina short reads (Ross et al., 2013). As such, all long reads underwent self-error correction before assembly using NextDenovo software (https://github.com/Nextomics/NextDenovo) with default parameters. After self-error correction, all reads were assembled into contigs using WTDBG v2.1 (Ruan and Li, 2019) with parameters -p 19 -k 1 -AS 4 -K 0.05 -s 0.5 to obtain raw genome assembly results. Then, we used Pilon (Walker et al., 2014) v1.21 with two iterations to polish the genome by filtered Illumina short reads (downloaded from NCBI). To assess the completeness of the final genome, several different strategies were used. BUSCO (Benchmarking Universal Single-Copy Orthologs) (Simao et al., 2015) was first employed using both Eukaryota and Metazoa core conserved genes as databases. To test the mapping ratio of Illumina reads, all filtered short reads were mapped to the assembled genome using BWA-MEM v0.7.12 (Li and Durbin, 2009).

### Genome Annotation

The genome annotation includes the repetitive elements annotation, gene annotation, and function annotation. For repetitive elements annotation, both tandem repeats and transposable elements (TEs) were determined for the Chinese mitten crab genome. For tandem repeats, Tandem Repeat Finder v4.09 (Benson, 1999) was used for tandem repeat annotation with default parameters. For TE annotation, we searched the TEs on both DNA level and protein level. The RepeatProteinMask (RM-BLASTX) software was used to search TEs with default parameters at the protein level. On DNA level, we used both the consensus sequences produced from the RepeatModeler software and the repbase library (downloaded from RepeatMsker website: http://www.repeatmasker.org/) employed this analysis by the RepeatMasker software (Bedell et al., 2000).

Based on the repeat-masked genome of the Chinese mitten crab, we applied *de novo* prediction, RNA-seq-based prediction, and homology-based prediction for gene structure annotation. For *de novo* prediction, the assembled Chinese mitten crab genome and RNA-seq data assembled transcripts were used for Augustus v2.5.5 (Stanke and Waack, 2003) training to obtain proper gene annotation results of Chinese mitten crab. We then used Augustus v2.5.5 (Stanke and Waack, 2003) for *de novo* prediction of coding genes using the previous training results of Chinese mitten crab. For homology-based prediction, to obtain high-quality gene annotation results, gene set from several species that have high-quality genome or have close relationship with Chinese mitten crab were downloaded, including *Bicyclus anynana* (GCF_900239965.1) (Nowell et al., 2017), *Bombus terrestris* (GCF_000214255.1) (Sadd et al., 2015), *Drosophila melanogaster* (GCA_000001215.4) (Adams et al., 2000), *Mus musculus* (GCF_000001635.26) (Gnerre et al., 2011), *Stegodyphus mimosarum* (GCA_000611955.2), *Penaeus vannamei* (GCA_003789085.1), *Mesobuthus martensii* (http://lifecenter.sgst.cn/main/en/scorpion.jsp)^1^ (Cao et al., 2013), *E. j. sinensis* (GigaDB: 100186) (Song et al., 2016), and *Tachypleus tridentatus* (GCA_004102145.1). For each species, for each gene we chose the longest transcript and used for *tblastn* (Altschul, 2012) search with an *e* value cutoff of 1e−5. The *tblastn* results were used for Genewise (Birney et al., 2004) analysis to obtain the final homology-based prediction results for each species. For RNA-seq prediction, the assembled transcripts were aligned against the genome using BLAT software (Kent, 2002) (identity >90% and coverage >90%), with PASA (Haas et al., 2003) used to filter the overlaps. EvidenceModeler (Haas et al., 2008) was used to integrate all the above results.

For functional annotation, we used InterProScan v4.8 (Zdobnov and Apweiler, 2001) for InterPro and Gene Ontology (GO) analyses. In addition, the Kyoto Encyclopedia of Genes and Genomes (KEGG) (Kanehisa, 2002), UniProt/SwissProt, and UniProt/TrEMBL databases were used for BLAST (Altschul, 2012) search with an *e* value cutoff set as 1e−5 and other parameters set to default.

### Gene Family Identification and Unique Gene Family Identification

For comparative genomics analysis, we used all the annotated genes in the Chinese mitten crab and five closely related species, including *Aedes aegypti* (GCF_002204515.2), *D. melanogaster* (GCA_000001215.4), *S. mimosarum* (GCA_000611955.2), *P. vannamei* (GCA_003789085.1), and *B. terrestris* (GCF_000214255.1). Genes with the longest transcripts of each species were chosen and used to run all to all BLAST (Altschul, 2012) search. Blastp were used and all the protein sequences of these genes were blasted to each other. The BLAST results were used as input for OrthoMCL (Li et al., 2003) and calculated the pairwise relationships of all genes. The reciprocal best similarity pairs (two-way BLAST best match) were considered as putative orthologous or paralogous genes, and the genes with no ortholog or paralog relationship were identified as unique genes. All the 1:1:1:1:1:1 single-copy genes identified among the six species were chosen and used for further gene family analysis. Besides, we also analyzed the unique gene family in the Chinese mitten crab genome relative to other five species for enrichment analysis. The enrichment analysis was performed using GOstat (Beissbarth and Speed, 2004). Fisher's exact test (chi-square test was used if gene numbers are large) was performed to judge whether the observed difference is significant or not. If the *P* value is less than 0.05, we considered that the difference is significant.

### Phylogenetic Relationship and Divergence Time Estimation

To determine the phylogenetic relationship of the Chinese mitten crab and other species (*A. aegypti*, *B. anynana*, *D. melanogaster*, *S. mimosarum*, and *P. vannamei*), we used the previous identified single-copy genes as input and utilized RAxML software (Stamatakis, 2014) with PROTGAMMAAUTO model. *S. mimosarum* (spider), as the only species in Arachnida among these six species, has the furthest relationship with the five Pancrustacea species. Therefore, *S. mimosarum* was used as the outgroup, and the single-copy genes of these six species were used for phylogenetic analysis using default parameters. After obtaining the tree of these six species, we performed the divergence time calculation. We employed MCMCTREE analysis in the PAML package (Yang, 1997) with nucleotide substitution model set as JC69 and other parameters set as default, with fossil records of these species from the TIMETREE website (http://www.timetree.org) used for calibration. The calibration data of *D. melanogaster* and *A. aegypti*, *P. vannamei* and *E. sinensis*, *P. vannamei* and *B. terrestris*, and *S. mimosarum* and *P. vannamei* were downloaded from the TIMETREE website.

### Gene Family Expansion and Contraction Analysis

We performed gene family expansion analysis of the six species using CAFE v4.0 along with the phylogenetic tree and divergence time. Considering that the expanded gene families in the Chinese mitten crab may have important functions, we conducted GO enrichment analysis subsequently. The enrichment analysis was performed using GOstat (Beissbarth and Speed, 2004) as previously described.

### Relative Evolution Rate Analysis

Different environments can place different survival pressures on different species. To determine the pressures on the above species, we performed relative evolution rate analysis using LINTRE version 1 (Takezaki et al., 1995) with the *tpcv* model, with spider as the outgroup species. We also used the Tajima's relative rate test. The spider were also used as outgroup and MEGA8 (Kumar et al., 1994) was used for analysis using default parameters.

## Results

### Assembly and Characterization of *E. j. sinensis* Genome

**Figure 1:**
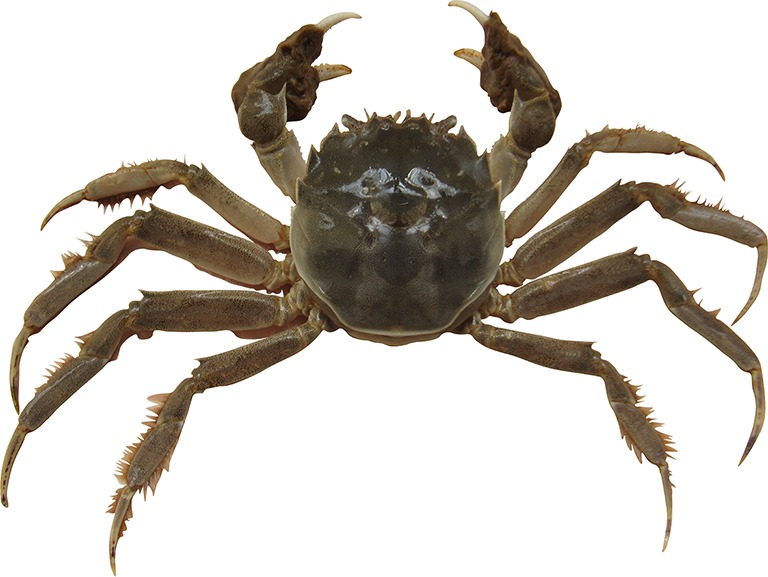
Chinese mitten crab, *Eriocheir japonica sinensis*.

**Figure 2:**
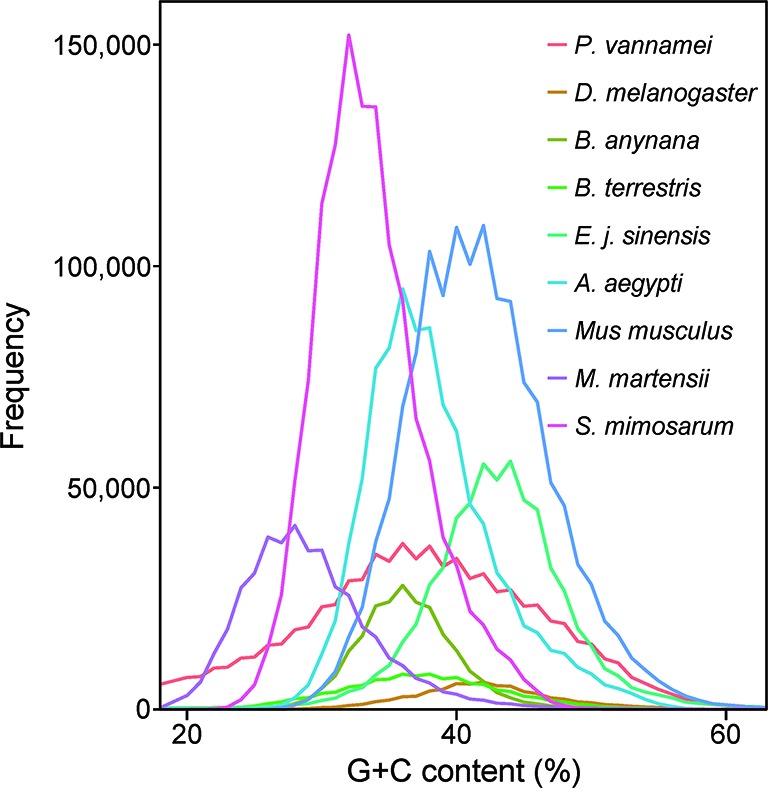
Guanine–cytosine (GC) content of Chinese mitten crab and other genomes. The GC content values were calculated using a sliding window method and the window size is 2 Kb. The whole-genome sequences including the coding region and non-coding region were used for analysis.

To construct a high-quality genome draft of the Chinese mitten crab, we extracted DNA from the muscle tissue of the crab (**Figure 1**) and constructed a high-quality Nanopore library for sequencing, which yielded a total of ~53.00 Gb filtered reads (**Table S1**). Using the downloaded 40.50× Illumina short reads (**Table S2**), the genome size of this crab was estimated about 1.19 Gb (**Figure S1**). We performed the contamination detection step and found that 4.39% reads of Illumina short reads and 1.30% Nanopore long reads had the best alignments to the microorganisms and not to other prokaryotes genome. These reads were considered as contamination reads and were all removed. Then, we used the retained Nanopore long reads and Illumina short reads to obtain the final assembled genome with N50 3.19 Mb, which is much better than the previously published Chinese mitten crab genome (Song et al., 2016) (**Table 1**). To assess the completeness of the final genome, several different strategies were used. BUSCO assessment showed that 94.00% of Eukaryota and 92.90% of Metazoa core conserved genes were found in the Chinese mitten crab genome (**Table 2**), indicating that most genes (at least over 92% genes were assembled) were assembled. All the filtered ~18-Gb RNA-seq data (**Table S3**) were assembled into transcripts with N50 1.75 Kb (**Table S4**). The mapping ratio of Illumina reads (98.82%), Nanopore reads (94.14%), and transcripts (81.32%) to our assembled genome indicated a good quality of the assembled genome (**Table 3** and **Table S5**). N50 of our assembled transcripts (1.76 Kb) and basic information compared to other species further indicated a good genome quality (**Table S6**). Besides, comparative guanine–cytosine (GC) content analysis of the genome with other species demonstrated that the GC content in the Chinese mitten crab (43%) was slightly higher than that of most related species (from 28% to 42%) (**Figure 2**).

**Table 1:** Statistics on assembled Chinese mitten crab genome and previously published Chinese mitten crab genome.

Term	Our genome		Previously published genome	
	Size (bp)	Number	Size (bp)	Number
N90	80,260	1,368	144	772,162
N80	305,684	511	405	220,601
N70	864,694	252	1,123	43,328
N60	1,666,934	149	42,141	3,605
N50	3,185,988	94	111,755	2,066
Max length	16,811,200	–	2,002,076	–
Total length	1,270,960,592	–	1,118,179,523	–
Number ≥ 100 bp	–	6,657	–	1,768,649
Number ≥ 10 kb	–	5,141	–	5,093

**Table 2:** Statistics on genome quality evaluation by BUSCO software.

Library	Eukaryota	Metazoa
Complete BUSCO (C)	285	909
Complete and single-copy BUSCO (S)	281	905
Complete and duplicated BUSCO (D)	4	4
Fragmented BUSCO (F)	5	25
Missing BUSCO (M)	13	44
Total BUSCO groups searched	303	978
Summary	94.00%	92.90%

**Table 3:** Statistics on Illumina short-read mapping ratio for assembled Chinese mitten crab genome.

Term	Length (bp)/Percentage
No. of total reads	1,177,643,075
No. of mapped reads	1,164,904,878
Mapped ratio (%)	98.82
No. of PE-mapped reads	836,906,878
PE-mapped ratio (%)	74.88
No. of SE-mapped reads	6,091,591
SE-mapped ratio (%)	0.55

PE-mapped represents reads mapped to genome as read pairs. SE-mapped represents reads mapped to genome as single reads.

### Genome Annotation

**Figure 3:**
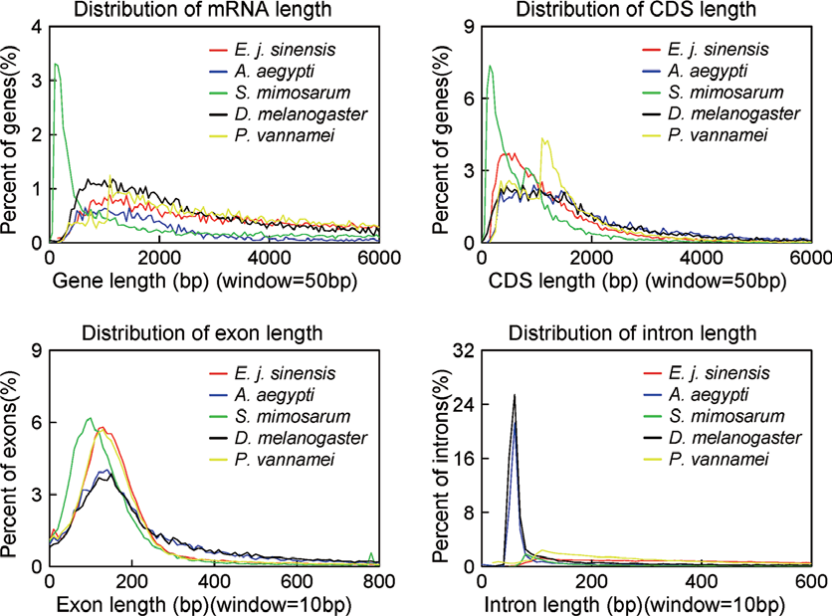
Quality comparison of protein-coding genes. The distribution of mRNA, CDS, exon, and intron among these species were shown. The species include *Eriocheir japonica sinensis*, *Aedes aegypti*, *Stegodyphus mimosarum*, *Drosophila melanogaster*, and *Penaeus vannamei*.

We conducted three parts of the genome annotation, including repeat annotation, gene annotation, and function annotation. In total, 780.68 Mb of repetitive sequences was identified, accounting for 61.42% of the assembled genome (**Table 4**). Among these repetitive sequences, 54.43% (~691.78 Mb) were TEs (**Table 5**). Based on the repeat-masked genome of the Chinese mitten crab, we applied gene structure annotation. And a total of 22,619 high-quality protein-coding genes were identified using the previously described annotation methods, including the *de novo* prediction, RNA-seq-based prediction, and homology-based prediction. The function annotation results showed that 92.43% of genes have orthology genes in the public database (**Table 6**). Comparison of the gene quality between the Chinese mitten crab and other published species, including *A. aegypti*, *S. mimosarum*, *P. vannamei*, and *D. melanogaster*, showed that the lengths of mRNA, coding sequences, exons, and introns were quite similar, suggesting that we acquired a reasonable gene set of the Chinese mitten crab (**Figure 3** and **Table S7**).

**Table 4:** Statistics on repetitive sequence annotation in Chinese mitten crab genome.

Type	Repeat size	% of genome
Tandem Repeat Finder	303,992,509	23.918327
RepeatMasker	167,854,132	13.206871
ProteinMask	111,728,104	8.790839
*De novo*	620,811,245	48.84583
Total	780,678,689	61.424303

**Table 5:** Statistics on transposable elements (TEs) in Chinese mitten crab genome.

Type	**Repbase TEs**		**TE proteins**		***De novo***		**Combined TEs**	
	**Length (bp)**	**% in genome**	**Length (bp)**	**% in genome**	**Length (bp)**	**% in genome**	**Length (bp)**	**% in genome**
DNA	92,713,892	7.29	3,569,566	0.28	58,584,466	4.61	149,193,019	11.74
LINE	12,182,661	0.96	97,531,450	7.67	164,787,780	12.97	201,524,402	15.86
SINE	70,066	0.0055	–	–	4,609,943	0.36	4,618,193	0.36
LTR	18,466,448	1.45	10,647,352	0.84	27,904,457	2.20	49,744,359	3.91
Other	78,097,956	6.14	3,723	0.00029	198,684,468	15.63	216,761,658	17.05
Unknown	96,240	0.0076	–	–	174,272,660	13.71	174,365,180	13.72
Total	167,854,132	13.21	111,728,104	8.79	620,811,245	48.85	691,775,509	54.43

**Table 6:** Statistics on functional annotation of protein-coding genes in Chinese mitten crab genome.

Term	Annotation number	Annotation percentage
GO	10,360	45.80
InterPro	14,698	64.98
KEGG	12,256	54.18
SwissProt	13,828	61.13
TrEMBL	20,492	90.60
Annotated	20,907	92.43
Unannotated	1,712	7.57
Total	22,619	–

### Orthologous Gene Identification

**Figure 4:**
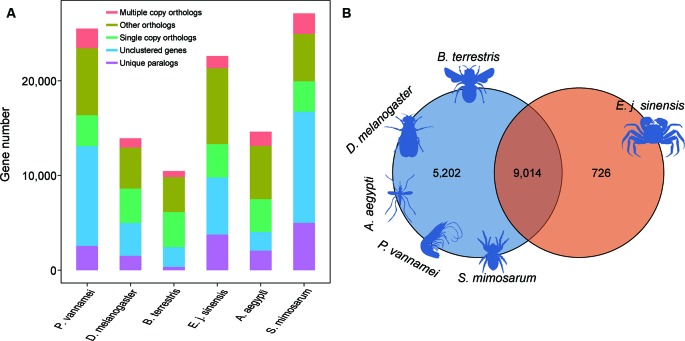
Gene family analysis of Chinese mitten crab. **(A)** Orthologous genes among species. **(B)** Unique and shared gene families in species.

For comparative genomics analysis, we used the annotated genes in the Chinese mitten crab and five other species, including *A. aegypti*, *D. melanogaster*, *S. mimosarum*, *P. vannamei*, and *B. terrestris*. We identified 2,324 single-copy genes among the six species, which were used for further gene family analysis (**Figure 4A**). We analyzed the unique gene family in the Chinese mitten crab genome relative to the other five species and finally identified 726 gene families for enrichment analysis (**Figure 4B**). Results indicated that they were mainly related to heterocyclic compound binding (adjusted P = 9.24e−45), structural constituent of cuticle (adjusted P = 2.99e−05), signaling receptor activity (adjusted P = 0.0021) processes, detection of stimulus (adjusted P = 3.43e−2), response to external stimulus (adjusted P = 4.78e−2), and response to abiotic stimulus (adjusted P = 4.78e−2) (**Table S8**), suggesting the unique immunity response or ability of Chinese mitten crab.

### Gene Family Expansion and Contraction Analysis

We performed gene family expansion analysis of the six species using CAFE v4.0 (De Bie et al., 2006) and found 603 expanded gene families in the Chinese mitten crab. As these gene families may have important functions in the crab, we conducted GO enrichment analysis. The results showed that the expanded genes were significantly enriched in nine GO terms (adjusted *P* < 0.05) (**Table S9**). Specifically, the expanded gene families were primarily related to structural constituent of cuticle (adjusted *P* = 1.11e−90), heterocyclic compound binding (adjusted *P* = 2.61e−30), ion binding (adjusted* P* = 5.39e−3), cellular metabolic process (adjusted *P* = 1.86e−2), and substrate-specific transporter activity (adjusted *P* = 3.21e−2) (**Table S9**). These categories are basic physiological processes, suggesting unique developmental and environmental adaptation of the Chinese mitten crab. We also checked the contracted gene families in Chinese mitten crab among these species. There are 868 contracted gene families, and the GO enrichment analysis showed that these genes are related to the macromolecular complex (adjusted *P* = 3.74e−24), non-membrane-bounded organelle (adjusted *P* = 7.62e−19), intracellular non-membrane-bounded organelle (adjusted *P* = 7.62e−19), intracellular organelle part (adjusted *P* = 2.54e−17), and organelle part (adjusted *P* = 2.54e−17) (**Table S10**).

### Phylogenetic Relationship and Divergence Time Estimation

**Figure 5:**
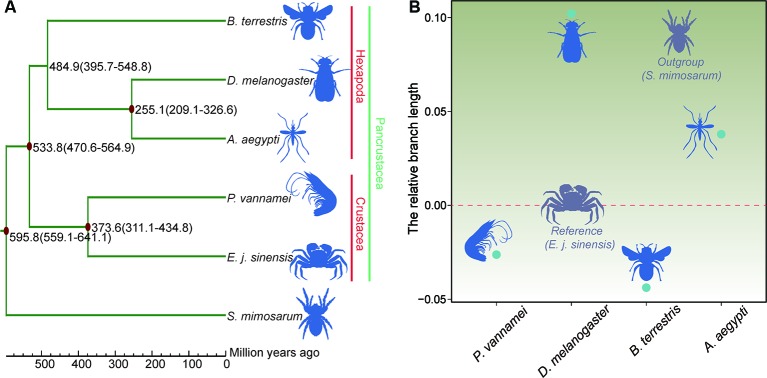
Phylogenetic relationships, divergence time, and evolution rate. **(A)** Phylogenetic relationship and divergence time of species. *Red dot* represents fossil record used here. **(B)** Relative evolution rate of species.

The phylogenetic relationship results showed that the Chinese mitten crab has a close relationship with *P. vannamei*, but a more distant relationship with *B. terrestris*, *D. melanogaster*, and *A. aegypti*. The five species of Pancrustacea formed two clades, i.e., Hexapoda and Crustacea (**Figure 5A**). The divergence time results showed that the Chinese mitten crab (Pleocyemata) and *P. vannamei* (Dendrobranchiata) diverged ~373.6 million years ago (Mya), and Crustacea diverged with the hexapods *B. terrestris*, *D. melanogaster*, and *A. aegypti* ~533.8 Mya (**Figure 5A**).

### Relative Evolution Rate Analysis

Different environments can place different survival pressures on different species. After the relative evolution rate analysis, the results indicated that the Chinese mitten crab had a faster evolution rate than the shrimp or bumblebee, but slower rate than the other three species (**Figure 5B** and **Table S11**), indicating that the Chinese mitten crab inhabits relatively stable environment and experiences little survival pressure.

## Conclusion

The size of the *E. j. sinensis* assembled genome was approximately 1.27 Gb, close to the estimated size (1.19 Gb), and the BUSCO results showed that 94.00% of the expected eukaryotic genes were completely present in the genome assembly. Comparative genomics analysis demonstrated that the Chinese mitten crab diverged from *P. vannamei* ~373.6 Mya, with a faster evolution rate than shrimp. The expanded genes of *E. j. sinensis* are basic physiological processes, suggesting unique developmental and environmental adaptation of the Chinese mitten crab. Compared with the previously published genome assembly of Chinese mitten crab, the assembly produced in this study has obvious improvement in genome continuity and gene completeness. We anticipate that the annotated high-quality genome of *E. j. sinensis* will promote research on its basic development and evolution and make substantial contributions to comparative genomic analyses of crustaceans.

## Data Availability Statement

The datasets generated for this study can be found in the BioProject database under the accession number PRJNA555707.

## Ethics Statement

This study was approved by the Animal Care and Use Committee, Jiangsu Provincial Key Laboratory of Coastal Wetland Bioresources and Environmental Protection, Yancheng Teachers University. The methods were carried out in accordance with approved guidelines.

## Author Contributions

YL, BT, DZ, GQ, and QW conceived and supervised the project. BT, QL, DZ, HZ, SS, and HS collected the samples. ZS, YS, FX, BG, and XW designed and carried out the experiments. ZhoW, HJ, YR, ZheW, and HL performed bioinformatics analyses. YL, BT, WW, and QQ wrote the manuscript. XL, SJ, and WJ helped perform analyses with constructive discussions. All authors have read and approved the final manuscript.

## Funding

This study was supported by the National Natural Science Foundation of China (31672267, 31640074), the Jiangsu Agriculture Science and Technology Innovation Fund (CX(18) 3027), the Natural Science Foundation of Jiangsu Province (BK20171276, BK20160444), “Qing Lan Project” of Daizhen Zhang, China Postdoctoral Science Foundation (2018M642105) and the National Key R&D Program of China (2018YFD0900201).

## Conflict of Interest

The authors declare that the research was conducted in the absence of any commercial or financial relationships that could be construed as a potential conflict of interest.

## Footnotes

1 http://lifecenter.sgst.cn/main/en/scorpion.jsp. Last accessed June 17, 2018.
