# Supplementary material for: High-Quality Genome Assembly of Eriocheir japonica sinensis Reveals Its Unique Genome Evolution
Source: Front Genet. 2020 Jan 17;10:1340. doi: 10.3389/fgene.2019.01340 (PMC6979310; doi:10.3389/fgene.2019.01340)

Table S1: The statistic of sequencing data on Nanopore platform. The reads with quality value Q > 7 and length > 1000 bp were considered. The sequencing depth was calculated by the assembled genome size.

| Cell | Average length (bp) | N50 length (bp) | Reads number | Total base(bp) | Sequence coverage (X) |
| --- | --- | --- | --- | --- | --- |
| 20190416-NPL0870-P1-E7-H7 | 20,216 | 25,986 | 430,529 | 8,703,671,243 | 7.30 |
| 20190417-NPL0870-P1-A9-D9 | 20,829 | 26,588 | 452,079 | 9,416,771,735 | 7.90 |
| 20190417-NPL0870-P2-E1-H1 | 20,673 | 26,595 | 209,730 | 4,335,870,431 | 3.64 |
| 20190419-NPL0870-P1-A1-D1 | 21,096 | 27,124 | 506,228 | 10,679,449,016 | 8.96 |
| 20190419-NPL0870-P1-A7-D7 | 21,916 | 28,298 | 432,204 | 9,472,490,907 | 7.95 |
| 20190419-NPL0870-P1-E1-H1 | 17,911 | 24,687 | 580,085 | 10,390,062,464 | 8.72 |
| Total/Average | 20,440 | 26,546 | 2,610,855 | 52,998,315,796 | 44.47 |

Table S2: The statistics of sequencing data download from NCBI. The sequencing depth was calculated by the assembled genome size.

| Library | Read number | Total bases (bp) | Sequencing platform | Sequencing depth (X) |
| --- | --- | --- | --- | --- |
| PRJNA305216 | 1,117,609,092 | ‬102,944,696,640 | Illumina | 81.00 |

Table S3: The statistics of RNA-seq data.

| Term | Read pairs | Total bases (bp) | Sequencing strategy |
| --- | --- | --- | --- |
| hepatopancreas | 53,224,948 | 7,662,952,512 | PE150 |
| heart | 44,187,186 | 6,362,954,784 | PE150 |
| muscle | 29,758,062 | 4,285,160,928 | PE150 |
| Total | 127,170,196 | 18,311,068,224 | - |

Table S4: The statistics of assembled transcripts in Chinese mitten crab.

| Term | Size (bp) | Number |
| --- | --- | --- |
| N90 | 300 | 106,987 |
| N80 | 495 | 68,998 |
| N70 | 783 | 45,648 |
| N60 | 1,193 | 30,561 |
| N50 | 1,758 | 20,518 |
| Max length (bp) | 32,665 | - |
| Total length (bp) | 145,019,472 | - |
| Total number (>100 bp) | - | 167,261 |
| Total number (>10 kb) | - | 290 |

Table S5: The statistics of the transcript mapping ratio on Chinese mitten crab genome.

| Mapped transcripts number | Total transcripts number | Mapping ratio |
| --- | --- | --- |
| 136,022 | 167,261 | 81.32% |

Table S6: The statistics of genome quality in these species.

| Species | Genome source | Assembly | Genome size | Scaffold N50 | Total scaffold number | BUSCO |
| --- | --- | --- | --- | --- | --- | --- |
| *Bombus terrestris* | NCBI | chromosome | 248,654,244 | 12,868,931 | 5,609 | 96.70% |
| *Mesobuthus martensii* | NCBI | contig | 925,546,267 | 45,228 | 92,408 | 58.40% |
| *Stegodyphusmimosarum* | NCBI | Scaffold | 2,738,704,917 | 480,636 | 68,653 | 88.50% |
| *Aedes aegypti* | NCBI | Chromosome | 1,870,673,364 | 310,827,022 | 6,534 | 99.40% |
| *Penaeus vannamei* | NCBI | Scaffold | 1,663,565,311 | 605,555 | 4,682 | 90.40% |
| *Bicyclus anynana* | NCBI | Scaffold | 475,399,557 | 638,282 | 10,800 | 97.70% |

Table S7: The statistics of gene stat in these species.

| species | Total gene number | Average gene length | Average CDS length | Average exon length | Average intron length |
| --- | --- | --- | --- | --- | --- |
| *E. j. sinensis* | 22,619 | 9246.24 | 1284.38 | 225.61 | 1692.71 |
| *A. aegypti* | 19,429 | 36511.32 | 2123.48 | 347.52 | 11913.30 |
| *S. mimosarum* | 27,135 | 22250.04 | 830.67 | 194.64 | 6259.34 |
| *D. melanogaster* | 18,240 | 5830.11 | 1954.96 | 371.74 | 1329.53 |
| *P. vannamei* | 25,527 | 8883.18 | 1520.72 | 256.73 | 1568.40 |

Table S8: The GO enrichment analysis of unique gene families in Chinese mitten crab genome.

| GO ID | GO Term | GO Class | *P*-value | Adjusted P-value |
| --- | --- | --- | --- | --- |
| GO:1901363 | heterocyclic compound binding | MF | 2.22E-46 | 9.24E-45 |
| GO:0097159 | organic cyclic compound binding | MF | 2.50E-46 | 9.24E-45 |
| GO:0032993 | protein-DNA complex | CC | 2.54E-13 | 4.69E-12 |
| GO:0042302 | structural constituent of cuticle | MF | 6.06E-06 | 2.99E-05 |
| GO:0038023 | signaling receptor activity | MF | 0.000837202 | 0.002065099 |
| GO:0004871 | signal transducer activity | MF | 0.002138332 | 0.004795047 |
| GO:0044764 | multi-organism cellular process | BP | 0.00273865 | 0.005790288 |
| GO:0044419 | interspecies interaction between organisms | BP | 0.00273865 | 0.005790288 |
| GO:0000150 | recombinase activity | MF | 0.008146745 | 0.015071478 |
| GO:0097367 | carbohydrate derivative binding | MF | 0.013730781 | 0.024782385 |
| GO:0051606 | detection of stimulus | BP | 0.019469538 | 0.034303471 |
| GO:0009605 | response to external stimulus | BP | 0.029088577 | 0.047834549 |
| GO:0009628 | response to abiotic stimulus | BP | 0.029088577 | 0.047834549 |

Table S9: The GO enrichment analysis of expanded gene families in Chinese mitten crab genome.

| GO ID | GO Term | GO Class | *P*-value | Adjusted P-value |
| --- | --- | --- | --- | --- |
| GO:0042302 | structural constituent of cuticle | MF | 2.05E-92 | 1.11E-90 |
| GO:1901363 | heterocyclic compound binding | MF | 1.34E-31 | 2.61E-30 |
| GO:0097159 | organic cyclic compound binding | MF | 1.45E-31 | 2.61E-30 |
| GO:0043167 | ion binding | MF | 0.00229487 | 0.005387956 |
| GO:0044237 | cellular metabolic process | BP | 0.008852077 | 0.018599933 |
| GO:0031409 | pigment binding | MF | 0.012708582 | 0.024509409 |
| GO:0022892 | substrate-specific transporter activity | MF | 0.017212822 | 0.032051462 |
| GO:0022857 | transmembrane transporter activity | MF | 0.018020653 | 0.032437175 |
| GO:0016740 | transferase activity | MF | 0.020433008 | 0.035592982 |

Table S10: The GO enrichment analysis of contracted gene families in Chinese mitten crab genome.

| GO ID | GO Term | GO Class | *P*-value | Adjusted P-value |
| --- | --- | --- | --- | --- |
| GO:0032991 | macromolecular complex | CC | 1.61E-26 | 3.74E-24 |
| GO:0043228 | non-membrane-bounded organelle | CC | 9.82E-21 | 7.62E-19 |
| GO:0043232 | intracellular non-membrane-bounded organelle | CC | 9.82E-21 | 7.62E-19 |
| GO:0044446 | intracellular organelle part | CC | 5.09E-19 | 2.54E-17 |
| GO:0044422 | organelle part | CC | 5.44E-19 | 2.54E-17 |
| GO:0000786 | nucleosome | CC | 4.86E-17 | 1.62E-15 |
| GO:0032993 | protein-DNA complex | CC | 4.86E-17 | 1.62E-15 |
| GO:0000785 | chromatin | CC | 6.43E-17 | 1.72E-15 |
| GO:0043226 | organelle | CC | 7.40E-17 | 1.72E-15 |
| GO:0043229 | intracellular organelle | CC | 7.40E-17 | 1.72E-15 |
| GO:0046982 | protein heterodimerization activity | MF | 1.45E-16 | 3.07E-15 |
| GO:0044427 | chromosomal part | CC | 4.05E-16 | 7.86E-15 |
| GO:0005694 | chromosome | CC | 1.06E-15 | 1.90E-14 |
| GO:0016459 | myosin complex | CC | 1.04E-14 | 1.74E-13 |
| GO:0015629 | actin cytoskeleton | CC | 1.52E-13 | 2.37E-12 |
| GO:0044424 | intracellular part | CC | 1.81E-12 | 2.63E-11 |
| GO:0016787 | hydrolase activity | MF | 5.87E-12 | 8.05E-11 |
| GO:0003774 | motor activity | MF | 3.90E-11 | 4.94E-10 |
| GO:0046983 | protein dimerization activity | MF | 4.03E-11 | 4.94E-10 |
| GO:0005622 | intracellular | CC | 4.53E-11 | 5.28E-10 |
| GO:0005623 | cell | CC | 1.93E-10 | 2.04E-09 |
| GO:0044464 | cell part | CC | 1.93E-10 | 2.04E-09 |
| GO:0008237 | metallopeptidase activity | MF | 2.36E-10 | 2.39E-09 |
| GO:0044430 | cytoskeletal part | CC | 7.87E-10 | 7.64E-09 |
| GO:0005856 | cytoskeleton | CC | 1.86E-09 | 1.73E-08 |
| GO:0017111 | nucleoside-triphosphatase activity | MF | 3.41E-07 | 3.06E-06 |
| GO:0016462 | pyrophosphatase activity | MF | 4.27E-07 | 3.68E-06 |
| GO:0016818 | hydrolase activity, acting on acid anhydrides, in phosphorus-containing anhydrides | MF | 5.09E-07 | 4.23E-06 |
| GO:0070011 | peptidase activity, acting on L-amino acid peptides | MF | 5.43E-07 | 4.36E-06 |
| GO:0016817 | hydrolase activity, acting on acid anhydrides | MF | 7.77E-07 | 5.99E-06 |
| GO:0008233 | peptidase activity | MF | 7.97E-07 | 5.99E-06 |
| GO:0003677 | DNA binding | MF | 1.16E-05 | 8.48E-05 |
| GO:0003824 | catalytic activity | MF | 0.00017137 | 0.001140833 |
| GO:0043234 | protein complex | CC | 0.000178235 | 0.001153574 |
| GO:0004601 | peroxidase activity | MF | 0.000459734 | 0.002818897 |
| GO:0016684 | oxidoreductase activity, acting on peroxide as acceptor | MF | 0.000459734 | 0.002818897 |
| GO:0006979 | response to oxidative stress | BP | 0.000552708 | 0.003302078 |
| GO:0016209 | antioxidant activity | MF | 0.000716161 | 0.004171635 |
| GO:0008270 | zinc ion binding | MF | 0.000890343 | 0.004939283 |
| GO:1901363 | heterocyclic compound binding | MF | 0.004687807 | 0.025003578 |
| GO:0097159 | organic cyclic compound binding | MF | 0.004721706 | 0.025003578 |
| GO:0042302 | structural constituent of cuticle | MF | 0.005268979 | 0.027281601 |
| GO:0046914 | transition metal ion binding | MF | 0.006330672 | 0.032066228 |
| GO:0004222 | metalloendopeptidase activity | MF | 0.007119528 | 0.035256498 |
| GO:0005634 | nucleus | CC | 0.008044382 | 0.038251858 |
| GO:0016747 | transferase activity, transferring acyl groups other than amino-acyl groups | MF | 0.00984449 | 0.044975809 |

Table S11: The relative evolutionary rate of *E. sinensis* and other species, as analyzed by Tajima’s test.

| Outgroup | Ingroup1 | Ingroup2 | Identical | Ingroup1  specific | Ingroup2  specific | Chi-score | P-value | Faster |
| --- | --- | --- | --- | --- | --- | --- | --- | --- |
| *S. mimosarum* | *E. sinensis* | *P. vannamei* | 343,835 | 38,207 | 33,308 | 335.60 | 0.000001 | *E. sinensis* |
| *S. mimosarum* | *E. sinensis* | *D. melanogaster* | 310,493 | 83,736 | 102,746 | 1937.88 | 0.000001 | *D. melanogaster* |
| *S. mimosarum* | *E. sinensis* | *B. terrestris* | 328,014 | 92,549 | 83,654 | 449.03 | 0.000001 | *E. sinensis* |
| *S. mimosarum* | *E. sinensis* | *A. aegypti* | 318,492 | 87,402 | 94,703 | 292.71 | 0.000001 | *A. aegypti* |

**Figure S1: Genomic survey of Chinese mitten crab genome.** The GenomeScope k-mer profile plot of the Chinese mitten crab dataset showing the fit of the GenomeScope model (black) to the observed k-mer frequencies (blue). The unusual peak of very high frequency k-mers (∼18× coverage) were determined to be highly enriched for organelle sequences


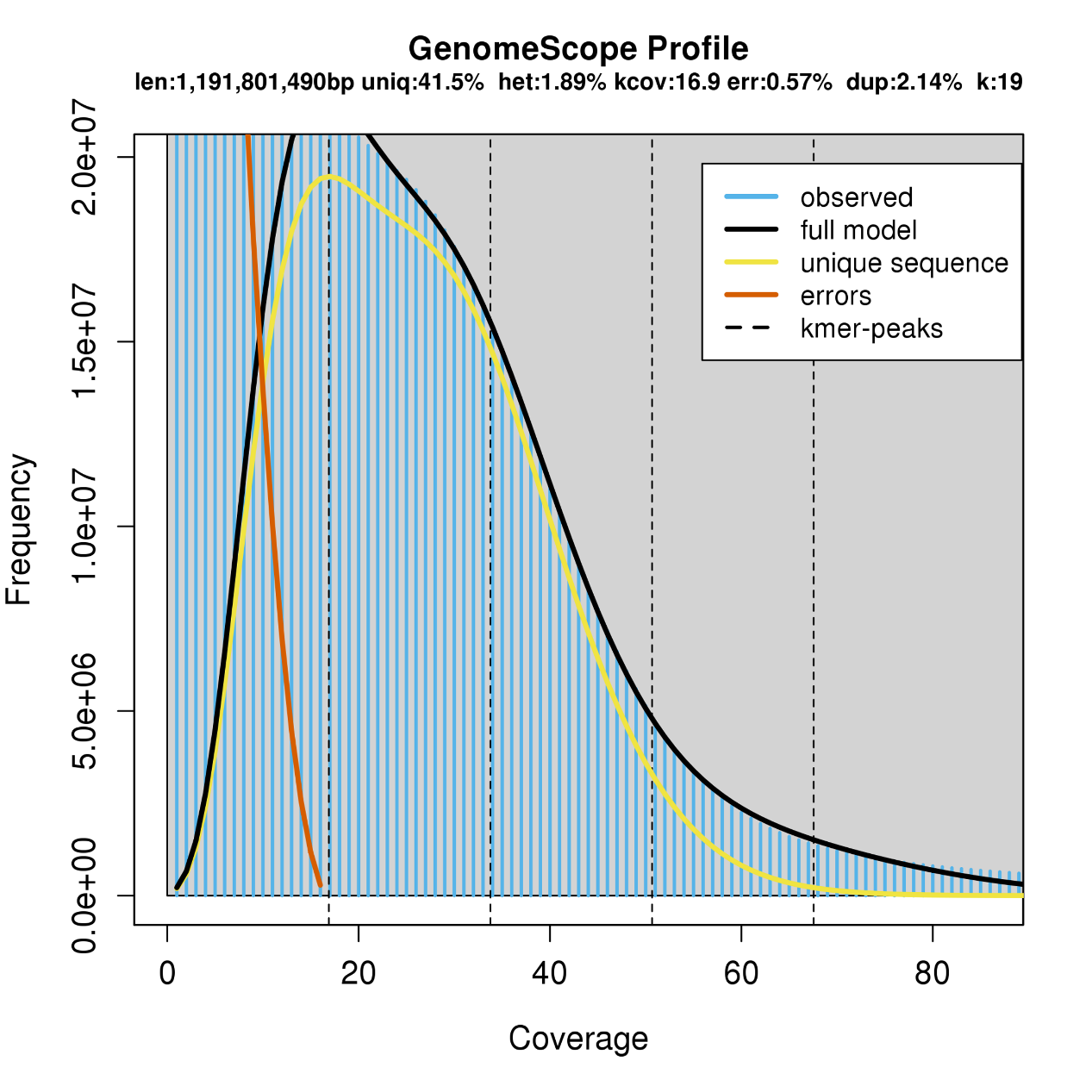

Supplement: Supplementary file 1 [file DataSheet_1.docx]
